# Isolated comminuted fracture of the cricoid cartilage and narrowing of the airway after a traumatic blunt injury of the neck: a case report

**DOI:** 10.1186/s12245-022-00459-9

**Published:** 2022-10-05

**Authors:** Saadat Mehrabi, Reza Hosseinpour, Mohammad Javad Yavari Barhaghtalab

**Affiliations:** Department of General Surgery, Shahid Beheshti Hospital, Yasuj University of Medical Sciences, Yasuj, Iran

**Keywords:** Cricoid cartilage, Fracture, Narrowing of the airway, Traumatic blunt injury, Neck

## Abstract

**Background:**

Blunt trauma to the anterior of the neck may compromise the vital structures like major blood vessels, trachea, larynx, pharynx, thyroid, spine, esophagus, and the cricoid. Laryngeal trauma is rare and accounts for 1% of all neck blunt traumas. Cricoid trauma is also very rare and accounts for half of the laryngeal traumas, and the diagnosis is frequently missed.

**Case presentation:**

A 43-year-old man, with blunt neck trauma after being hardly hit by a crane lifting hook, was referred to the Shahid Beheshti Hospital. The patient complained of dysphonia (hoarseness) and dyspnea. The CT scans showed a comminuted fracture of the left anterior arch of the cricoid cartilage with left-sided mucosal thickening, inflammation, and edema which was extended to the glottis, causing a narrowing of the airway. Direct fiber-optic laryngoscopy revealed swelling and congestion in the epiglottis and swelling at the level of the left vocal cord.

**Conclusion:**

This case report highlights the conservative treatment of isolated cricoid cartilage fracture in the setting of low-energy blunt trauma. The patient was clinically stable and treated conservatively with oxygen therapy and silence therapy (complete silence).

## Background

Blunt trauma to the anterior of the neck may compromise the vital structures like major blood vessels, trachea, larynx, pharynx, thyroid, spine, esophagus, and the cricoid [1]. Laryngeal trauma is rare and accounts for 1% of all neck blunt traumas. Cricoid trauma is also very rare and accounts for half of the laryngeal traumas, and the diagnosis is frequently missed [2]. In this study, a case of isolated cricoid fracture with airway inflammation and narrowing after a blunt neck trauma is presented.

## Case presentation

**Fig. 1:**
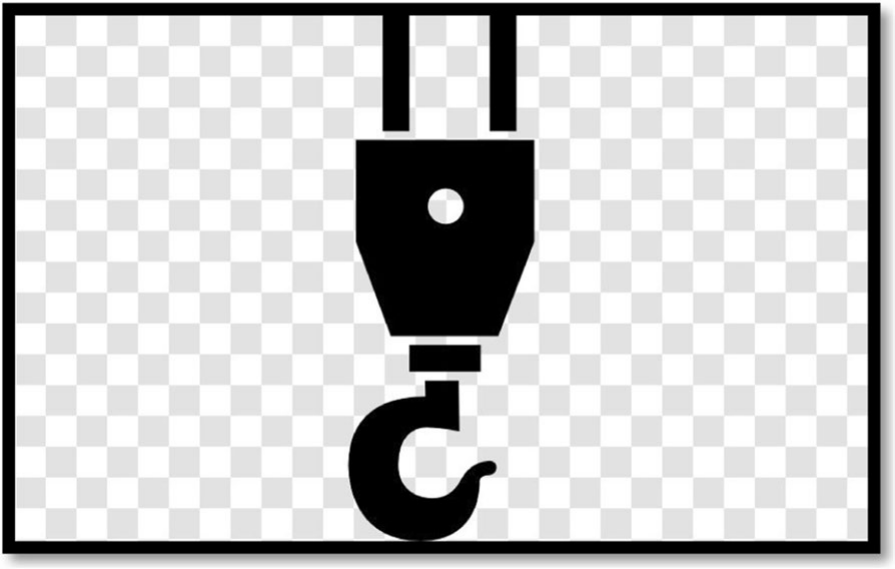
A crane lifting hook

A 43-year-old man, with blunt neck trauma after being hardly hit by a crane lifting hook (Fig. 1), was referred to the Shahid Beheshti Hospital Emergency Department (affiliated to Yasuj University of Medical Sciences, Yasuj, Iran). At the initial visit, the cervical collar was fixed first. The patient was evaluated in the primary survey and findings were as below:

A: The airway system was open without any tracheal deviation, and the respiratory rate was 18 breaths/min with the oxygen saturation value of 98% estimated by pulse oximetry, while oxygen was administered at a rate of 3 L/min.

B: Breathing was spontaneous without decreased breathing sound in bilateral auscultation of the lungs (no pneumothorax), but there was stridor, and the chest had bilateral symmetrical expansion, and there was no or subcutaneous emphysema.

**Fig. 2:**
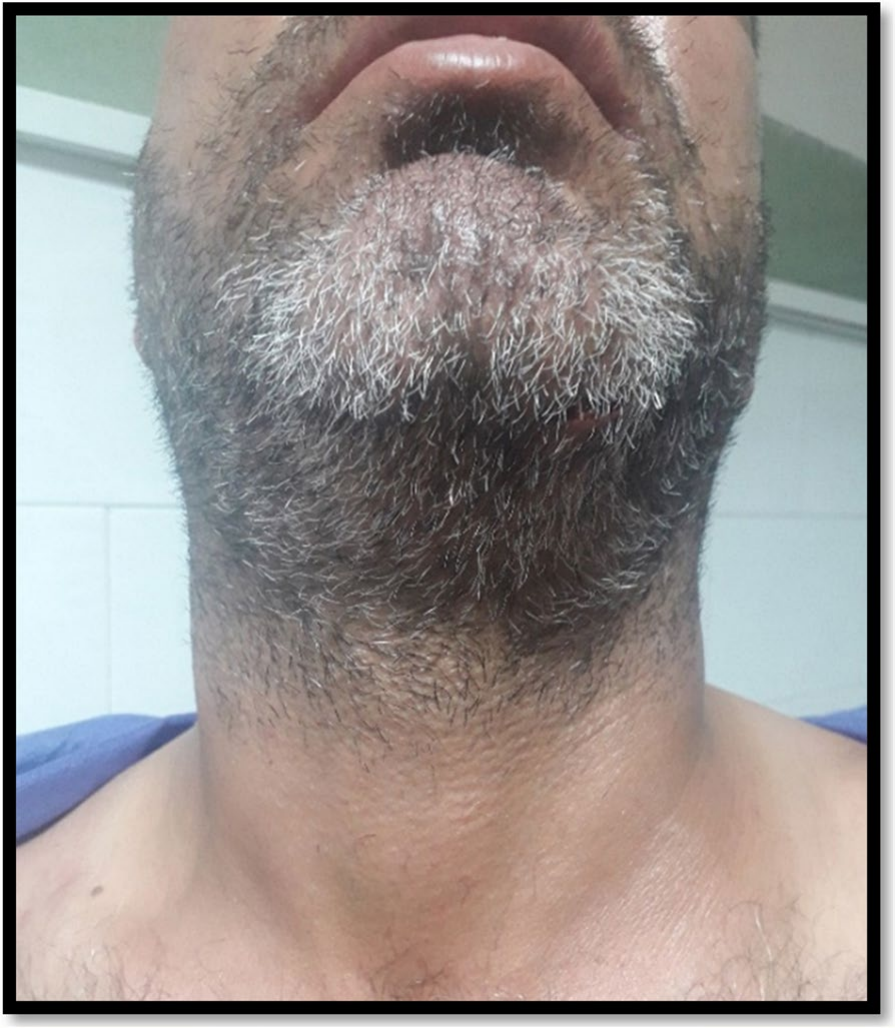
No ecchymosis, bruising, hematoma, and external bleeding in the trauma site at the neck

C: His blood pressure and pulse rate were 100/70 mm Hg and 92 beats/min respectively. Carotid pulses were present bilaterally. There was no ecchymosis, bruising, hematoma, and external bleeding in the trauma site at the neck (Fig. 2).

D: The patient was alert with the Glasgow Coma Scale/Score (GCS) of 15/15, and there was no cervical spine pain and tenderness, while the cervical collar was fixed.

E: The patient was exposed while kept warm, and there were no other findings in the physical examinations.

There was no abnormality in the simple radiography of the cervical spine. Color Doppler sonography (CDS) of the carotid and vertebral arteries and jugular vein showed normal flow velocity and spectral waveforms in the common carotid artery (CCA), internal carotid artery (ICA), external carotid artery (ECA), and vertebral arteries, and there was normal flow in both internal jugular veins.

**Fig. 3:**
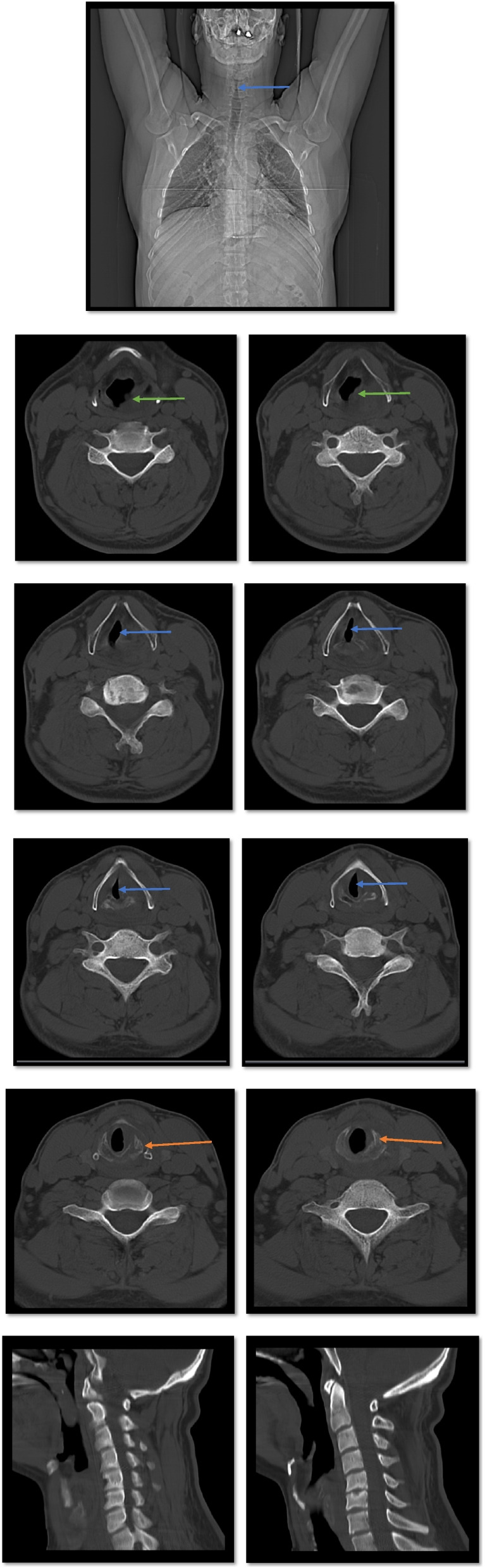
Spiral neck CT scan with IV contrast showing cricoid cartilage fracture and narrowing of the airway (axial and sagittal views). Green arrows show mucosal thickening, inflammation, and edema extended to the glottis, blue arrows show narrowing of the airway, and orange arrows show the cricoid fracture

While the patient’s hoarseness and dyspnea got worse with time, neck and chest CT scans were performed to rule out laryngeal and other chest trauma. The CT scans showed no pathology in the chest but comminuted fracture of the left anterior arch of the cricoid cartilage with left-sided mucosal thickening, inflammation, and edema which was extended to the glottis, causing a narrowing of the airway (transverse inner diameter of the cricoid = 3.7 mm) (Fig. 3).

The cervical spine had no fracture. Direct fiber-optic laryngoscopy revealed swelling and congestion in the epiglottis and swelling at the level of the left vocal cord. The arterial oxygen saturation value was sustained at > 98% by administration of oxygen at a rate of 3–5 L/min with the use of a mask. As the patient’s vital sign was stable, and the arterial oxygen saturation value through the pulse oximetry did not decrease with time, orotracheal intubation and tracheotomy were not performed. The patient tolerated the treatment and had good intervention adherence. There were no adverse and unanticipated events during the study.

Dysphonia and dyspnea alleviated gradually, and on the 4th day after the admission, the patient was discharged. The patient visited again on the 7th day after the discharge, and there was no dyspnea but very mild and fading dysphonia (clinically improved). The patient was optimistic about his well-being and coming back to his work again in the future. One of the limitations of this study was that we could not follow the patient with the next-up laryngoscopy.

## Discussion

Larynx trauma is rare, but it may cause airway obstruction [2]. Isolated cricoid fractures are very rare and life threatening because the cricoid is the only circumferential cartilage in the larynx and is essential for the stability and integrity of the airway [3]. For a surgeon, the most important factor for the diagnosis of a laryngeal injury is having a high index of suspicion [2, 4]. The three clinical findings representing laryngeal fractures are hoarseness, subcutaneous emphysema, and palpable fracture [2, 5]. The patient’s inability to tolerate the supine position seen in severely injured patients should consider immediate tracheotomy without performing a laryngoscopic examination [6].

In patients with an obstructed airway or severe respiratory distress, recommended airway control measurement is orotracheal intubation. Emergency tracheostomy is indicated if intubation is unsuccessful [2, 5]. In patients with edema, hematomas, nondisplaced fractures, exposed cartilage, cord immobility, or complete laryngotracheal separation, immediate tracheotomy followed by additional studies or exploration as early operative management is beneficial [6]. Surgical cricothyroidotomy is not preferred but may be a lifesaving option [2, 5]. In this study, as the patient had a stable vital sign, and had no respiratory distress, and there was no hematoma, orotracheal intubation and operative measurements like tracheostomy were not done, but the treatment was successfully done only with supportive care, including continuous monitoring of clinical symptoms and physical examinations at short intervals.

## Conclusion

This case report highlights the conservative treatment of isolated cricoid cartilage fracture in the setting of low-energy blunt trauma. The patient was clinically stable and treated conservatively with oxygen therapy and silence therapy (complete silence).

## Data Availability

The datasets used and/or analyzed during the current study are available from the corresponding author on reasonable request.

## Abbreviations

GCS: Glasgow Coma Scale/Score
CDS: Color Doppler sonography
CCA: Common carotid artery
ICA: Internal carotid artery
ECA: External carotid artery
CT scan: Computerized tomography

## References

[1] Shenoy MS, Mathew V, Mathews I (2021). Isolated cricoid fracture and thyroid hematoma in blunt injury of the neck. Indian J Otolaryngol Head Neck Surg.

[2] Oh JH, Min HS, Park TU, Lee SJ, Kim SE (2007). Isolated cricoid fracture associated with blunt neck trauma. Emerg Med J..

[3] Falcone TE, Schwartz MA, Lee AS, Anderson T (2017). Conservative treatment of isolated cricoid cartilage fractures from blunt trauma. JAMA Otolaryngol Head Neck Surg.

[4] Pekcevik Y, Cukurova I, Ulker C (2013). Cricoid and thyroid cartilage fracture, cricothyroid joint dislocation, pseudofracture appearance of the hyoid bone: CT, MRI, and laryngoscopic findings. J Acad Emerg Med.

[5] Fitzsimons MG, Peralta R, Hurford W (2005). Cricoid fracture after physical assault. J Trauma..

[6] Fuhrman GM, Stieg FH, Buerk CA (1990). Blunt laryngeal trauma: classification and management protocol. J Trauma.

